# Artificial Intelligence (AI) in Radiology: A Deep Dive Into ChatGPT 4.0's Accuracy with the American Journal of Neuroradiology's (AJNR) "Case of the Month"

**DOI:** 10.7759/cureus.43958

**Published:** 2023-08-23

**Authors:** Pokhraj P Suthar, Avin Kounsal, Lavanya Chhetri, Divya Saini, Sumeet G Dua

**Affiliations:** 1 Department of Diagnostic Radiology and Nuclear Medicine, Rush University Medical Center, Chicago, USA; 2 Department of Clinical Nutrition, Rush University Medical Center, Chicago, USA; 3 Department of Public Health, Johns Hopkins Bloomberg School of Public Health, Baltimore, USA

**Keywords:** large language models (llms), accuracy, chat gpt, neuroradiology, artificial intelligence in radiology

## Abstract

The advent of artificial intelligence (AI), particularly large language models (LLMs) such as ChatGPT 4.0, holds significant potential in healthcare, specifically in radiology. This study examined the accuracy of ChatGPT 4.0 (July 20, 2023, version) in solving diagnostic quizzes from the American Journal of Neuroradiology's (AJNR) "Case of the Month." We evaluated the diagnostic accuracy of ChatGPT 4.0 when provided with a patient's history and imaging findings weekly over four weeks, using 140 cases from the AJNR "Case of the Month" portal (from November 2011 to July 2023). The overall diagnostic accuracy was found to be 57.86% (81 out of 140 cases). The diagnostic performance varied across brain, head and neck, and spine subgroups, with accuracy rates of 54.65%, 67.65%, and 55.0%, respectively. These findings suggest that AI models such as ChatGPT 4.0 could serve as useful adjuncts in radiological diagnostics, thus potentially enhancing patient care and revolutionizing medical education.

## Introduction

Advancements in artificial intelligence (AI), specifically in the domain of large language models (LLMs), are ushering in a new era in the field of healthcare [1]. Of these LLMs, ChatGPT, with its versions 3.5 and 4.0, stands out as one of the most widely used and accessible models [2]. In particular, ChatGPT 4.0 presents a significant stride forward, having been trained with a substantial dataset of radiology articles as part of the GPT-4 training process [3]. This innovative integration has prompted an outpouring of research investigating the potential applications of ChatGPT in the assessment and educational sectors. Among the most promising of these explorations are studies examining the role of ChatGPT in evaluating various quizzes and exams [4-6]. This reflects the broader potential of LLMs in enhancing medical diagnostics and patient care and revolutionizing medical education and examination systems. This research aims to ascertain the precision of ChatGPT in resolving diagnostic quizzes from the "Case of the Month" feature of the American Journal of Neuroradiology (AJNR). By doing so, we aim to assess its potential as a supportive tool in radiological diagnostics and to address the unique contribution it makes to the existing body of knowledge. By measuring ChatGPT's performance in these complex clinical scenarios, we aim to explore its potential as a decision-support tool in diagnostic radiology. This is a crucial step toward integrating AI-driven models into healthcare, effectively augmenting medical professionals' decision-making processes and potentially improving patient outcomes.

## Materials and methods

The objective of this study is to determine the accuracy of the GPT-4-based ChatGPT (July 20, 2023, version) in solving diagnostic quizzes presented in the AJNR's "Case of the Month." The aim is to evaluate the accuracy of GPT-4-based ChatGPT when provided with history and imaging findings weekly over four weeks. As this study was based on publicly available literature, there was no requirement for ethical approval. The design of this study adhered to the Standards for Reporting Diagnostic Accuracy (STARD) guidelines [4]. A total of 141 cases are available on the AJNR's "Case of the Month" portal from November 2011 to July 2023 [7]. From those, we did not include one case from February 2018 in which imaging findings were not provided in the caption to avoid bias. Ultimately, we included 140 cases in our analysis, from which we extracted patient history and image findings. Certain cases might incorporate pathology slides, histology findings, and diagnoses in the captions. In such cases, we only considered the histology findings and deliberately excluded the diagnoses mentioned in the captions. This was done to minimize the chance of artificially inflating the positive analysis results generated by ChatGPT. The descriptions of the AJNR's "Case of the Month" were meticulously drafted by the submitting author and subsequently reviewed and approved by the editors of the AJNR. Our team, comprised of three fellowship-trained neuroradiologists, has meticulously evaluated all the cases, and, to our knowledge, we have not identified any discrepancies or errors in the descriptions provided by the AJNR's "Case of the Month" portal.

**Figure 1:**
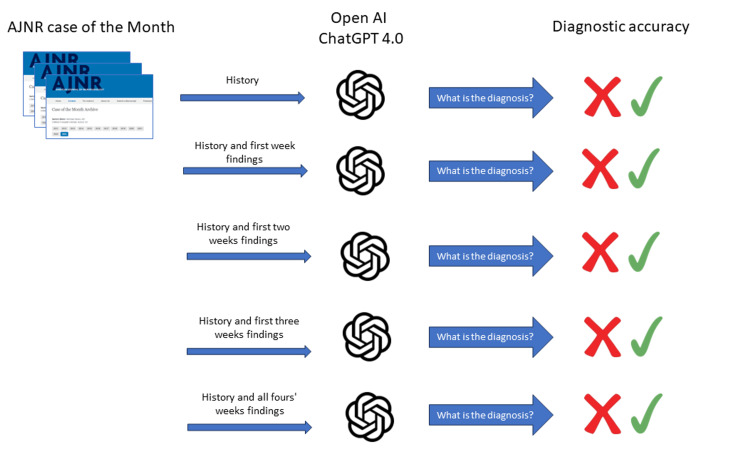
Study overview. This study evaluates the accuracy of the GPT-4 based ChatGPT (July 20, 2023, version) in solving diagnostic quizzes presented in the American Journal of Neuroradiology's (AJNR) "Case of the Month."

Given that ChatGPT cannot process images directly, it was provided with descriptions of image findings from the captions in the AJNR's "Case of the Month." These descriptions were furnished weekly, enabling ChatGPT to generate differential diagnoses. Initially, the clinical history was provided, after which imaging findings were introduced stepwise each week over four weeks, as is the practice in the AJNR "Case of the Month." This structured approach allowed us to evaluate how effectively ChatGPT could utilize this incremental information to reach a diagnostic conclusion. "We used a unique question to ask ChatGPT: 'What is the diagnosis?'" A visual summary of the study can be found in** **Figure 1. The radiologists independently reviewed and confirmed the alignment of the diagnoses generated by ChatGPT with those previously published. The validity of differential diagnoses produced by ChatGPT was further assessed by the same radiologists, utilizing a five-point Likert scale. On this scale, a score of 1 indicated a "highly improbable" differential diagnosis, while a score of 5 denoted it as "highly probable." The scores ranged from a minimum of 3.0 in the lower quartile to a maximum of 5.0 in the upper quartile. The Likert scores of 4.0 and 5.0 were considered satisfactory performance.

All the relevant data were compiled in a Microsoft Excel spreadsheet, enabling us to evaluate the accuracy as a percentage. Additionally, the cases were categorized into three subgroups: brain, head and neck, and spine. Separate analyses were conducted for each subtype to assess the accuracy of ChatGPT within these specific domains.

## Results

**Figure 2:**
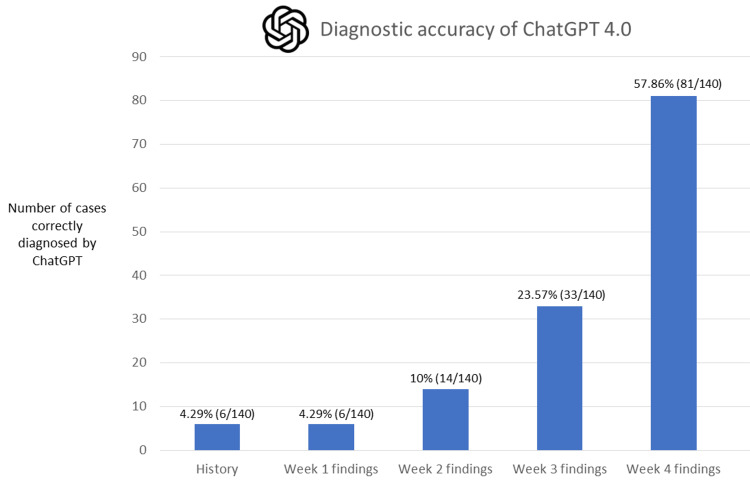
Study outcome overview. This study demonstrates how the diagnostic accuracy of ChatGPT increases incrementally with the stepwise addition of historical data and weekly findings. The overall diagnostic accuracy of ChatGPT for resolving the AJNR's "Case of the Month" is 57.86% (81 out of 140 cases).

The diagnostic performance of ChatGPT varied based on the volume of information provided. It achieved a diagnostic accuracy of 4.29% (six out of 140 cases) when only the patient history provided by the submitter was given. The accuracy was 4.29% (six out of 140 cases) when the history and the imaging findings from the first week were combined. When the history and imaging findings from the first two weeks were provided, the accuracy rose to 10% (14 out of 140 cases). Further adding the imaging findings from the third week led to an accuracy of 23.57% (33 out of 140 cases). Ultimately, combining patient history with imaging findings collected over the entire four-week span resulted in ChatGPT achieving a diagnostic accuracy of 57.86% (81 out of 140 cases). This figure signifies the overall diagnostic accuracy of ChatGPT for resolving the AJNR's "Case of the Month." The accuracy metrics are displayed in Figure 2.

**Figure 3:**
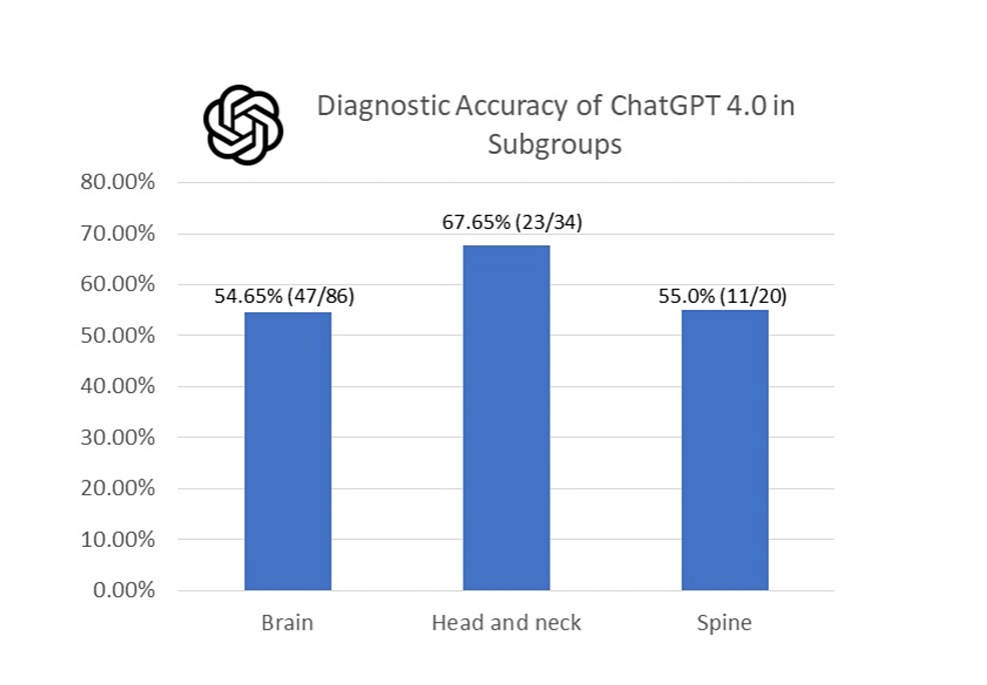
Study outcome overview. This study demonstrates how the diagnostic accuracy of ChatGPT varied across the subgroups. For the brain category, ChatGPT demonstrated an accuracy of 54.65% (47 out of 86 cases). In the head and neck category, the accuracy climbed to 67.65% (23 out of 34 cases), while in the spine category, the system delivered an accuracy of 55.0% (11 out of 20 cases).

The comprehensive diagnostic proficiency of ChatGPT varied across the subgroups. For the brain category, ChatGPT demonstrated an accuracy of 54.65% (47 out of 86 cases). In the head and neck category, the accuracy climbed to 67.65% (23 out of 34 cases), while in the spine category, the system delivered an accuracy of 55.0% (11 out of 20 cases) (Figure 3).

## Discussion

Evolution of AI in language processing

The domain of AI has experienced significant strides recently, specifically in the areas of natural language processing (NLP) and LLMs [1]. The advent of advanced NLP/LLM models has fundamentally altered the dynamic of human-computer interaction by endowing machines with the ability to understand and respond to intricate language nuances. As these AI systems continue to mature and become increasingly intelligent, they offer the potential to reshape various industries and significantly improve the quality of life for many individuals worldwide [1]. The emergence of ChatGPT, particularly the GPT-4 architecture, has opened the doors to various applications and exploration possibilities in the research world [3,8].

ChatGPT's comparative performance in medical examinations

Strong et al. conducted a study comparing the performance of chatbots and medical students on free-response clinical reasoning tests. The fourth model of the chatbot surpassed the performance of first- and second-year students in these exams and showed marked enhancement compared to model 3.5 [9]. A transition from the original chatbot to an updated version was assessed, and it surpassed the passing benchmark for multiple-choice queries, mimicking the US Medical License Examinations [10]. GPT-4 showcased significant advancements compared to GPT-3.5 in both professional and academic metrics, notably achieving higher scores in the uniform bar examination (rising from the 10th to the 90th percentile) and registering a boost of over 30% in the US Medical Licensing Examination [11,12]. A chatbot outperforming students in clinical reasoning tests presents difficulties in assessing students' clinical abilities. While closed-book exams might serve as an interim measure, they do not truly reflect the synthesis of information from varied sources. The trend in medical evaluations is moving toward open-book styles, evident from the inclusion of UpToDate in the American Board of Internal Medicine recertification exams [13].

Public reception and ethical discourse on LLM

Over recent years, the ethical ramifications of LLMs have sparked growing scholarly discourse [14]. The conversation broadened to the general public with OpenAI's launch of ChatGPT on November 30, 2022. Released as a research preview to gather user feedback and understand its capabilities and limitations, the chatbot saw over a million users experimenting with it in the subsequent week [15].

ChatGPT's performance in radiology diagnostics

The overall performance of ChatGPT in deciphering the AJNR's "Case of the Month" - utilizing both patient histories and detailed findings over four weeks - has an accuracy rate of 57.86%. This accomplishment is noteworthy when compared to a study by Ueda et al., which demonstrated ChatGPT's diagnostic efficacy as 54% for the "Diagnosis Please" quizzes, employing both patient histories and authors' descriptions of imaging findings [4]. Furthermore, in the study by Ueda et al., ChatGPT showcased an impressive 87% accuracy rate without choices, and a remarkable 97% accuracy rate with choices, after excluding 16 image-based NEJM quizzes [6].

Exploration of GPT-4 in radiology reporting

In the June 2023 issue of Radiology, Sun et al. explored the capabilities of GPT-4 in producing radiology report impressions [16]. While their study draws a noteworthy comparison between impressions crafted by radiologists and those by GPT-4, it also subtly suggests the promising future of AI models in radiology. Elkassem et al. suggested that LLMs such as ChatGPT can enhance radiology reporting and boost patient interaction. While they can help with precision and speed, they also come with challenges such as biases, data privacy issues, and ethical concerns [17]. Bhayana et al. have highlighted ChatGPT's significant advancements in radiology, noting that GPT-4 outperformed GPT-3.5 in a radiology board test by over 10% [18]. Its improved reasoning capabilities are evident in radiological contexts, and its understanding of specific terminology is commendable. However, its sporadic errors and "hallucinations" raise questions about its dependability. These rapidly evolving models suggest promising avenues for specialized applications in radiology.

Educational and operational implications of ChatGPT

Educators can now effortlessly utilize ChatGPT for designing quizzes. Even though ChatGPT, sometimes known as chatgtp or chat openai, is changing how many operate, it might appear somewhat complex to some teachers [19]. ChatGPT could be a transformative tool in radiology, potentially easing the workload of healthcare professionals by providing rapid and reliable diagnostic results. The value of this AI system is accentuated in scenarios where there is a shortage of radiologists, an issue prevalent in many parts of the world. ChatGPT assists in streamlining diagnostic procedures by efficiently filling this gap and contributing to a more efficient healthcare delivery system. As such, the advent of this technology may pave the way for more strategic resource allocation and improve patient outcomes in the long run [20,21].

Study limitations

Still, this study was not without its limitations. These encompass ChatGPT's limitation in directly analyzing images and the possibility that post-analysis conclusions by authors may have boosted the noted superior performance. There was no evaluation of diagnostic performance based solely on imaging findings without the accompanying clinical history, even though such scenarios frequently arise in real-world practice.

Ethical and societal challenges of AI in radiology

Integrating AI models such as ChatGPT into radiology raises ethical and societal challenges. Biases in training data can skew diagnoses, potentially marginalizing specific groups. Patient data, while essential for AI processing, pose significant privacy concerns, from maintaining anonymity to securing data storage and transfer. In terms of patient-doctor dynamics, an over-reliance on AI might depersonalize care, alter trust dynamics, and blur error attribution lines. The professional realm may witness potential skill atrophy and job displacement fears among radiologists. Ethical quandaries also arise around accountability and the transparency of AI decisions, especially when models operate as "black boxes." Lastly, on a broader scale, there is concern about healthcare inequalities due to AI's potential high costs and the need for cultural sensitivity in global deployments. Balancing these issues is vital to harness AI's benefits while safeguarding the essence of patient-centric care.

## Conclusions

The findings of this study underscore the evolving role of large language models such as ChatGPT 4.0 in the realm of radiology. With an overall accuracy of 57.86% in solving the AJNR's "Case of the Month" diagnostic quizzes, ChatGPT 4.0 showcases the potential to be a valuable adjunct in radiological diagnostics. Its varied performance across brain, head and neck, and spine subgroups highlights areas of strength as well as domains that may require further optimization. While human expertise remains irreplaceable, the integration of AI tools such as ChatGPT could complement radiologists' efforts, potentially leading to expedited diagnostics and improved patient care. As the technological landscape in healthcare continues to evolve, it will be imperative to further investigate and refine the synergistic relationship between human intelligence and AI to harness the best outcomes for patient care and medical education.
